# Learnable Feature Disentanglement with Temporal-Complemented Motion Enhancement for Micro-Expression Recognition

**DOI:** 10.3390/e28020180

**Published:** 2026-02-04

**Authors:** Yu Qian, Shucheng Huang, Kai Qu

**Affiliations:** 1 School of Computer, Jiangsu University of Science and Technology, Zhenjiang 212100, China; 231210704105@stu.just.edu.cn; 2 College of Naval Coast Defense Army, Navy Aviation University, Yantai 264000, China

**Keywords:** feature disentanglement, micro-expression recognition, motion, optical flow

## Abstract

Micro-expressions (MEs) are involuntary facial movements that reveal genuine emotions, holding significant value in fields like deception detection and psychological diagnosis. However, micro-expression recognition (MER) is fundamentally challenged by the entanglement of subtle emotional motions with identity-specific features. Traditional methods, such as those based on Robust Principal Component Analysis (RPCA), attempt to separate identity and motion components through fixed preprocessing and coarse decomposition. However, these methods can inadvertently remove subtle emotional cues and are disconnected from subsequent module training, limiting the discriminative power of features. Inspired by the Bruce–Young model of facial cognition, which suggests that facial identity and expression are processed via independent neural routes, we recognize the need for a more dynamic, learnable disentanglement paradigm for MER. We propose LFD-TCMEN, a novel network that introduces an end-to-end learnable feature disentanglement framework. The network is synergistically optimized by a multi-task objective unifying orthogonality, reconstruction, consistency, cycle, identity, and classification losses. Specifically, the Disentangle Representation Learning (DRL) module adaptively isolates pure motion patterns from subject-specific appearance, overcoming the limitations of static preprocessing, while the Temporal-Complemented Motion Enhancement (TCME) module integrates purified motion representations—highlighting subtle facial muscle activations—with optical flow dynamics to comprehensively model the spatiotemporal evolution of MEs. Extensive experiments on CAS(ME)^3^ and DFME benchmarks demonstrate that our method achieves state-of-the-art cross-subject performance, validating the efficacy of the proposed learnable disentanglement and synergistic optimization.

## 1. Introduction

Facial expressions are important social cues in human communication, reflecting one’s emotional state. Based on differing physiological mechanisms, they can be categorized into macro-expressions (MaEs) and micro-expressions (MEs) [1]. Unlike MaEs, MEs are involuntary, unconscious emotional leaks. Consequently, they are regarded as genuine emotional responses, driving applications in lie detection, psychological evaluations, and security screenings. However, the intrinsic subtlety and shortness of MEs hinder the ability of models to capture effective representations [2], and the entanglement of motion and identity-specific features in facial representations significantly affects model generalization.

Early MER methods relied primarily on traditional machine learning techniques and manual feature engineering. Local Binary Patterns (LBPs) [3] and its variations [4,5,6] are representative examples. Researchers also explored optical flow features to capture facial muscle movement, using techniques like Main Directional Mean Optical Flow (MDMO) [7] and optical strain descriptors [8]. However, these methods struggle to capture fine-grained facial dynamics and generalize well across different subjects.

MER has significantly advanced with the application of deep learning, enabling more accurate and robust feature extraction. Early research employed smaller, more concise networks for feature extraction [9,10], whereas subsequent studies explored more sophisticated methodologies, including ROI-based techniques [4,11], attention mechanisms [12,13,14], Transformers [15,16], and multi-branch fusion structures [17,18,19], which enhanced the modeling of intricate dependencies and the integration of complementary information. Despite their achievements, these techniques often fail to account for identity-specific features within facial representations, which hampers cross-subject generalization. Recent works, such as DiSTLBP-RIP [20] and PSN [21], have addressed this by using RPCA to separate identity and motion components. However, these approaches treat disentanglement as a fixed preprocessing step, disconnecting it from the subsequent training process and limiting overall performance.

At the cognitive level, the Bruce–Young model of facial cognition [22] proposes that facial identity and expression are processed via functionally independent routes. This is supported by neuropsychological patients who can no longer recognize familiar faces yet retain the ability to interpret facial expressions, and vice versa. Such double dissociation suggests distinct neural mechanisms for identity and emotion processing, providing biological plausibility for our feature-disentanglement design. Meanwhile, recent studies like $D^{2}CA$ [23] in facial Action Unit detection demonstrate that learnable disentanglement techniques [24] can dynamically decouple task-relevant and task-irrelevant features during training. Building on these insights, we introduce learnable feature disentanglement to MER for the first time. Our key contribution lies in learning to disentangle identity-invariant motion features from subject-specific appearance, while enhancing subtle facial motion cues and modeling temporal continuity to yield a more expressive and robust motion representation.

The main contributions of our work include the following:

- Based on the Bruce–Young model of facial cognition, we propose a learnable feature disentanglement paradigm for MER, realized through a plug-and-play Disentangled Representation Learning (DRL) module that can be integrated into arbitrary MER networks. It dynamically isolates identity-invariant motion features from appearance information, effectively mitigating feature entanglement.
- We design a Temporal-Complemented Motion Enhancement (TCME) module that enriches purified spatial motion features with optical-flow-based temporal cues, enabling a comprehensive and fine-grained modeling of micro-expression spatiotemporal patterns.
- To ensure effective and unified optimization of the entire network, we introduce a Synergistic Disentanglement Objectives (SDO) scheme that jointly optimizes soft orthogonality, reconstruction, cycle, consistency, identity-aware contrastive, and emotion classification losses. Combined with our multi-stage training strategy, we effectively separate emotion-related motion features and individual-specific identity characteristics while avoiding the loss of subtle motion cues of MEs.
- Extensive experiments on CAS(ME)^3^ and DFME benchmarks demonstrate state-of-the-art cross-subject performance, validating the effectiveness and generalization of our learnable disentanglement and temporal enhancement framework.

## 2. Related Works

### 2.1. Micro-Expression Recognition

Micro-expression recognition (MER) has attracted growing interest from the computer vision community, undergoing significant methodological evolution. Early research relied on traditional machine-learning algorithms that required time-consuming hand-crafted features. A typical example is LBP [3], which leverages local texture information and has inspired multiple variants, such as DCP-TOP (Dual-cross Patterns from Three Orthogonal Planes) [6], LBP-SIP (LBP with Six Intersection Points) [5], and LBP-TOP [4]-these descriptors are customized to model spatiotemporal information for ME analysis [25]. Meanwhile, optical flow [26] was adopted to track facial movements; for instance, Liu et al. [7] developed MDMO by integrating face alignment into the optical flow framework. However, these traditional methods rely on manual feature engineering. Their dependence on hand-crafted descriptors and fixed parameters often leads to limited generalization capability.

The rise of deep learning has significantly advanced MER, with diverse network designs proposed to tackle the subtleties of MEs. Some works adopt ROI-based designs tailored to MEs’ local characteristics: Wang et al. [4] employed the Facial Action Coding System [27] (FACS) to partition the face into 16 ROIs and extract dynamic-texture descriptors within each region. Similar methodologies were further investigated in later studies [11,28]. Nonetheless, these defined ROI methods may overlook subtle expressions in non-predefined areas and may not be adaptable to individual facial variances. Other works leverage attention mechanisms for adaptive region selection: Chen et al. [12] designed a spatiotemporal attention module to find important regions across frames, and this line of research was furthered in [13,14,29]. However, attention-based methods might not be able to distinguish the difference between identity-related and expression-related facial regions, which could lead to poor feature representations. Transformer-based architectures have been developed to enhance benchmark performance by capturing long-range dependencies among facial regions [15,16,30]. However, their considerable complexity and the necessity for substantial data present challenges for the small-sample MER task.

Recognizing that single-modality strategies might overlook supplementary information, numerous studies have explored multi-branch fusion structures for MER. Sun et al. [17] suggested a dual-stream network jointly processing RGB and optical flow to capture motion and texture information. Liu et al. [18] proposed a multi-stream CNN that processes optical flow from onset-apex and apex-offset frame pairs. Kumar et al. [19] combined landmark-based and texture features using dual-stream graph attention networks. The excellent performance of these methods demonstrates that integrating complementary modalities could enhance complicated ME modeling. Inspired by these works, our approach uses a dual-branch architecture, but it differs as it separates identity from motion features before fusion.

However, existing deep learning approaches share a critical limitation: emotion-relevant motion features are entangled with person-specific identity characteristics, significantly hindering cross-subject generalization in MER tasks. To address this issue, several studies have attempted to decouple identity cues from expression dynamics before feeding data into subsequent recognition modules. For instance, DiSTLBP-RIP [20] employs RPCA [31] to decompose each video into a low-rank identity subspace and a sparse motion residual, then performs integral-projection-based LBP encoding only on the motion residual, thereby preventing the classifier from exploiting static facial structure. Similarly, PSN [21] adopts the same RPCA decomposition, discarding the low-rank component and feeding the sparse motion part to a shallow CNN to force the network to learn only identity-invariant micro-movements. However, a key limitation of these approaches is that they treat RPCA as a fixed, pre-processing denoising step. This creates a disconnect between the disentanglement process and the subsequent model training, which limits the overall performance from the outset. Unlike such prior work, our network embeds disentanglement into end-to-end training: identity and motion features are jointly optimized through a multi-loss combination, enabling the network to self-calibrate the separation and supply purified motion features to subsequent network structures.

Beyond feature and network design innovations, researchers also began addressing computational efficiency issues in ME analysis. Liong et al. [32] observed that complete ME sequences introduce unnecessary redundancy, impeding focus on discriminative features. They suggested obtaining dynamic features from the onset and apex frames to make computing faster and better at telling features apart. Many people have used the keyframe-based approach in their networks [9,10,33], which shows that it works well for choosing strategic frames. Inspired by these works, we also adopt a dual-frame input of the onset frame and apex frame instead of the whole video sequence.

### 2.2. Feature Disentanglement

A fundamental challenge in MER is the entanglement of facial identity with expression dynamics. Due to the scarcity of ME datasets, models tend to overfit to subject-specific characteristics rather than learning generalized expression patterns, leading to poor performance on unseen individuals [34]. This persistent challenge has prompted scholars to employ representation learning methodologies to differentiate task-relevant attributes from distractions.

The primary objective of disentangled feature learning [24] in visual representation is to segregate independent sources of variance within seen data into distinct dimensions. Some recent disentanglement algorithms [35,36] use clear supervisory signals to help with decomposition, while others [37,38] strive to automatically uncover hidden independent pieces in pure data distributions without supervision.

Supervision-guided disentanglement techniques require human annotation of variation factors, which makes practical applications scale poorly. Using fully annotated training samples, Aberman et al. [35] deconstruct 2D pose sequences into motion, skeleton, and viewpoint components in their motion retargeting framework. Reed et al. [36] employ before-and-after image pairs to learn visual analogy representation spaces. These methods produce semantically explicit and controlled representations, but they need labels across varied datasets, which makes them too expensive and unsuitable for real-world use.

Unsupervised disentanglement techniques, such as those in [37,38], discern independent variation components from data structures without the use of labels. This is attractive: no costly, time-consuming annotations. However, this requires balancing disentanglement with representation quality, as excessive disentanglement may harm feature learning. We obtain this balance by optimizing a multi-loss combination together. The reconstruction task works as an implicit quality monitor, making sure the images are high-fidelity while keeping features separate, all without any manual labels.

To summarize, existing MER research has advanced in feature engineering (e.g., LBP variants and optical flow) and network design (e.g., attention, Transformers, and multi-branch structure), but key limitations persist: most methods fail to fundamentally resolve the high coupling of identity and motion features, restricting cross-subject generalization. To address this problem, we design a learnable feature disentanglement network for MER. Specifically, we use unsupervised disentangled representation learning to extract pure motion structure features, which are further complemented with optical-flow-derived temporal cues to enhance subtle motion perception and achieve more comprehensive spatiotemporal modeling.

## 3. Proposed Method

**Figure 1:**
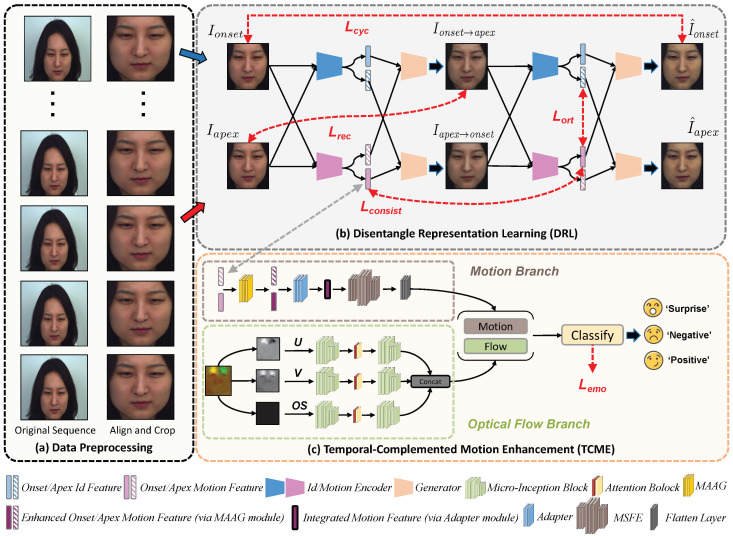
The pipeline of our proposed MER network. It consists of the Data Preprocessing module, as shown in (**a**), the Disentangle Representation Learning (DRL) module, as shown in (**b**), and the Temporal-Complemented Motion Enhancement (TCME) module, as shown in (**c**). In the Data Preprocessing module, it takes the original ME sequence as input, performs alignment and cropping on the frames, and outputs the processed onset and apex frame images. The DRL module utilizes dual lightweight encoders (Identity Encoder $E_{\mathrm{id}}$ and Motion Encoder $E_{\mathrm{motion}}$) with the same architecture to extract identity-related and motion-related features from the preprocessed images, and then reconstructs images through generators *G* to enhance feature disentanglement. The TCME module takes the disentangled motion representations and optical flow as inputs, uses two parallel branches to process motion and flow-related information, respectively, fuses these features, and finally realizes the classification of MEs. The bottom legend maps each icon to its corresponding module or representation.

**Figure 2:**
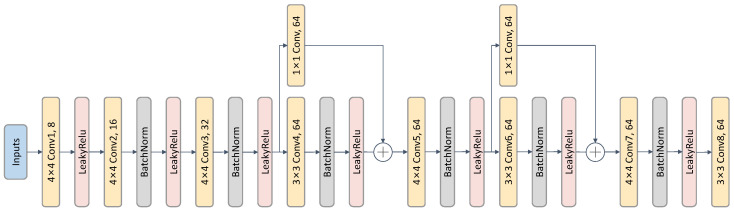
Encoder architecture. For identity features and motion structure features, we employ an identical architecture.

Our proposed method tackles the MER task using a unified framework, as depicted in Figure 1. The network has two main components: (1) the Disentangle Representation Learning (DRL) module, which disentangles features and reconstructs face images, as shown in Figure 1b, and (2) the Temporal-Complemented Motion Enhancement (TCME) module, which extracts complementary spatial motion features and optical flow temporal features and fuses them to complete the classification task, as shown in Figure 1c. We further introduce Synergistic Disentanglement Objectives (SDO) as the training core. This unified loss scheme integrates soft orthogonality, reconstruction, cycle, consistency, identity-aware contrastive, and emotion classification losses to jointly guide DRL’s feature disentanglement and TCME’s spatiotemporal modeling, addressing traditional methods’ insufficient decoupling issue.

### 3.1. Disentangle Representation Learning Module

At the heart of our MER architecture is a DRL module that employs two identical encoders and a generator, both specifically designed to capture and reconstruct subtle facial motion patterns. The encoders extract motion and identity features from onset–apex frame pairs; once these two distinct feature components have been explicitly disentangled and separated from each other, they are passed to a generator for face image reconstruction. During the initial reconstruction process, the onset identity features are combined with the apex motion features to generate a synthetic apex frame, and vice versa. To improve feature quality and ensure consistency, a cycle generation process is introduced: the synthetic images are re-encoded, and the motion and identity features from the same frame are combined to complete the reconstruction task, forming a closed loop to strengthen the disentanglement goal.

#### 3.1.1. Identity and Motion Encoder

As shown in Figure 2, we design a unified encoder architecture tailored for micro-expression representation learning. Specifically, the encoder adopts a lightweight eight-layer convolutional hierarchy capable of capturing subtle facial motion variations while preserving stable identity characteristics. Through progressive spatial downsampling and localized feature refinement, it produces 256-channel discriminative representations and retains multi-scale intermediate features via skip connections to support high-fidelity reconstruction in the generator.

Before encoding, we apply the nose-tip-based facial alignment strategy from [39] to eliminate global head motion and ensure spatial consistency across subjects. All facial inputs are thus normalized to a unified resolution of $R^{256\times 256\times 3}$.

The encoder adopts a progressive downsampling architecture. In the first stage, conv1–conv3 reduce spatial resolution while increasing channel depth (8 → 16 → 32), expanding the receptive field and capturing low-level facial texture details. In the second stage, conv4 and conv6 employ 3 × 3 convolutions to enhance local feature discrimination, enabling the network to represent both subtle muscle activations and identity-preserving structural information. Finally, conv7–conv8 produce a compact 256-dimensional latent representation that serves as the semantic embedding for subsequent decoding while maintaining computational efficiency.

To prevent the loss of fine-grained details during repeated downsampling, the encoder incorporates a targeted skip-connection strategy. Features after conv3 preserve local textural cues, whereas features after conv5 convey mid-level structural information. These multi-scale descriptors are passed to the generator, which helps maintain both motion- and identity-related information that could otherwise vanish near the bottleneck. This design also facilitates smoother gradient propagation and improves the reconstruction fidelity of subtle facial variations, often only a few pixels in amplitude.

#### 3.1.2. Face Reconstruction Generator

**Figure 3:**
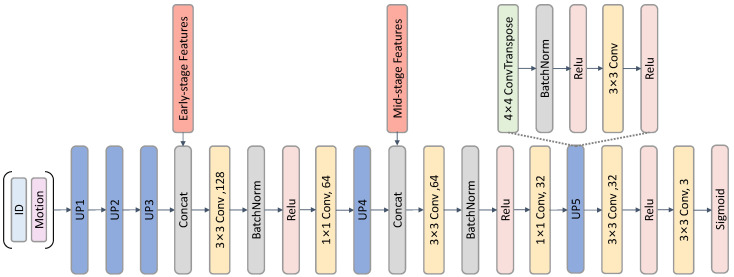
Generator architecture with skip connection fusion. The generator reconstructs facial images through cross-combination of identity and motion features, incorporating multi-scale skip features from the encoder for detail preservation.

As illustrated in Figure 3, we develop a reconstruction-oriented generator specifically designed for micro-expression feature disentanglement. Unlike conventional decoder designs that simply reverse the encoder, our generator enables cross-combination reconstruction (e.g., pairing onset identity with apex motion to synthesize an apex frame), which is essential to disentangle motion from appearance and enforce identity-invariant encoding.

The generator adopts a five-stage progressive upsampling pipeline (UP1–UP5) that transforms the concatenated identity and motion embeddings into the image space. Each stage integrates 4 × 4 transposed convolutions (stride 2) for spatial resolution recovery and 3 × 3 convolutional refinement blocks (stride 1) to suppress checkerboard artifacts and improve spatial coherence. Batch Normalization and ReLU activations are applied between layers to stabilize optimization.

To prevent the loss of essential micro-movement cues during decoding, we implement a dual-scale skip fusion mechanism that integrates complementary encoder characteristics into the generator. Early-stage skip features keep the fine-grained textures of local muscles, while mid-level skip features provide structural facial context. These two levels of information are selectively fused at appropriate decoding stages to enhance reconstruction fidelity.

The final output layer maps the features to the 3-channel RGB space using a sigmoid activation, constraining pixel intensities to [0,1] for stable reconstruction. By training the generator using cross-combination inputs, we enforce structural consistency from identity cues and dynamic consistency from motion cues, enabling it to serve as a functional supervision bridge for learning high-quality disentangled motion representations.

### 3.2. Synergistic Disentanglement Objectives

To jointly optimize the entire network, we design a unified training scheme called Synergistic Disentanglement Objectives (SDO). SDO integrates multiple complementary constraints, including soft orthogonality, reconstruction, cycle, consistency, identity-aware contrastive, and emotion classification losses, to jointly guide feature disentanglement and temporal modeling. This multi-constraint learning strategy effectively separates identity features from motion cues, while ensuring that subtle facial movements are preserved, thus preventing the loss of important motion information that could arise from excessive disentanglement.

#### 3.2.1. Soft Orthogonality Constraint

Our goal is to obtain pure spatial motion features that are decoupled from identity-related features, while ensuring that these two feature types remain distinct in the representation space. To achieve this, we introduce a soft orthogonality constraint. The soft orthogonality constraint “disrupts” the coupling between identity and motion structures; at the same time, the strength of this “disrupts” is limited by weights at different training stages, thus avoiding the loss of subtle motion details that are critical for accurate MER:

(1) $$L_{\mathrm{ort}}=E\left[\cos^{2}\left(E_{\mathrm{id}}\left(I\right),E_{\mathrm{motion}}\left(I\right)\right)\right]$$

$L_{\mathrm{ort}}$ represents soft orthogonality loss, $E_{\mathrm{id}}\left(\cdot \right)$ extracts individual-specific identity features, $E_{\mathrm{motion}}\left(\cdot \right)$ captures individual-independent facial movement features, and *I* represents the input facial expression frame. The cosine similarity cos(·,·) assesses the connection between identity and motion characteristics, whereas E[·] represents the batch expectation operation. We enforce the soft orthogonality constraint to decouple identity and motion representations by minimizing squared cosine similarity.

#### 3.2.2. Reconstruction Constraint

We combine identity and motion structure features from onset and apex frames to generate synthetic images $I_{onset\to apex}$ that closely match the apex frame, and vice versa. Since there is no identity difference between the onset and apex frames, we consider the synthetic image recreated in this manner to be nearly identical to the original. We compute per-pixel and perceptual losses for the synthetic image, which enables the network to differentiate between identity and motion structure information. This reconstruction process ensures that subtle motion cues are preserved during the disentanglement, preventing the loss of critical motion details that could occur with excessive separation. The reconstruction loss calculation procedure:

(2) $$\begin{array}{cc} I_{\mathrm{onset}\to \mathrm{apex}} & =G\left(\left[E_{\mathrm{id}}\left(I_{\mathrm{onset}}\right)⊕E_{\mathrm{motion}}\left(I_{\mathrm{apex}}\right)\right]\right), \end{array}$$

(3) $$\begin{array}{cc} I_{\mathrm{apex}\to \mathrm{onset}} & =G\left(\left[E_{\mathrm{id}}\left(I_{\mathrm{apex}}\right)⊕E_{\mathrm{motion}}\left(I_{\mathrm{onset}}\right)\right]\right) \end{array}$$

(4) $$L_{\mathrm{rec}}=L_{L1}+\lambda \cdot L_{\mathrm{perceptual}}$$

(5) $$\begin{array}{cc} L_{L1} & =E[|I_{\mathrm{onset}\to \mathrm{apex}}-I_{\mathrm{apex}}\left|\right]+E[|I_{\mathrm{apex}\to \mathrm{onset}}-I_{\mathrm{onset}}\left|\right] \end{array}$$

(6) $$\begin{array}{cc} L_{\mathrm{perceptual}} & =\sum_{i=1}^{5}w_{i}\cdot {∥VGG_{i}\left(I_{\mathrm{onset}\to \mathrm{apex}}\right)-VGG_{i}\left(I_{\mathrm{apex}}\right)∥}_{2}^{2}\\ \; & \;+∥VGG_{i}\left(I_{\mathrm{apex}\to \mathrm{onset}}\right)-VGG_{i}\left(I_{\mathrm{onset}}\right){∥}_{2}^{2} \end{array}$$

where $L_{\mathrm{rec}}$ denotes the reconstruction loss, G(·) represents the generator. Perceptual loss is calculated using multi-layer VGG-19 features, with $VGG_{i}\left(\cdot \right)$ extracting features from the *i*-th layer, $w_{i}$ representing layer-specific weights [1/2.6,1/16,1/3.7,1/5.6,1.0], λ=0.05 balancing the contribution, and ${∥\cdot ∥}_{2}^{2}$ representing the squared L2. The reconstruction loss enhances the encoder’s understanding of discriminative features and the decoder’s reconstruction capability by improving both onset to apex and apex to onset reconstruction quality.

#### 3.2.3. Cycle Reconstruction Constraint

We re-extract features from $I_{\mathrm{onset}\to \mathrm{apex}}$ and $I_{\mathrm{apex}\to \mathrm{onset}}$ using the encoders. By concatenating feature pairs corresponding to either the onset or apex frames, we generate ${\hat{I}}_{onset}$ and ${\hat{I}}_{apex}$ nearly indistinguishable from their original counterparts. This process further validates and strengthens the decoupling quality achieved by the network. The formula is as follows:

(7) $$\begin{array}{cc} {\hat{I}}_{onset} & =G\left(\left[E_{\mathrm{id}}\left(I_{\mathrm{onset}\to \mathrm{apex}}\right)⊕E_{\mathrm{motion}}\left(I_{\mathrm{apex}\to \mathrm{onset}}\right)\right]\right), \end{array}$$

(8) $$\begin{array}{cc} {\hat{I}}_{apex} & =G\left(\left[E_{\mathrm{id}}\left(I_{\mathrm{apex}\to \mathrm{onset}}\right)⊕E_{\mathrm{motion}}\left(I_{\mathrm{onset}\to \mathrm{apex}}\right)\right]\right) \end{array}$$

$L_{\mathrm{cyc}}$ denotes the cycle reconstruction loss that validates the feature disentanglement quality by ensuring the twice-reconstructed images remain faithful to their original counterparts. The calculation method for $L_{\mathrm{cyc}}$ is identical to that of $L_{\mathrm{rec}}$ described above.

#### 3.2.4. Consistency Constraint

As the identity information of the synthetic image $I_{\mathrm{onset}\to \mathrm{apex}}$ comes from $I_{\mathrm{onset}}$, the extracted features from $I_{\mathrm{onset}\to \mathrm{apex}}$ by $E_{\mathrm{id}}$ should be highly similar to those of $I_{\mathrm{onset}}$. While $I_{\mathrm{onset}\to \mathrm{apex}}$ is formed by injecting motion features from $I_{\mathrm{apex}}$, $E_{\mathrm{motion}}$ should extract features similar to those of $I_{\mathrm{apex}}$. The same is true for $I_{\mathrm{apex}\to \mathrm{onset}}$. We define consistency loss $L_{\mathrm{consist}}$ under the above perspectives:

(9) $$L_{\mathrm{consist}}=L_{\mathrm{onset}-\mathrm{id}}+L_{\mathrm{onset}-\mathrm{motion}}+L_{\mathrm{apex}-\mathrm{id}}+L_{\mathrm{apex}-\mathrm{motion}}$$

Each loss term in Equation (9) measures the consistency between the feature pairs described above, computed as the mean squared loss between the original feature $z_{x}$ and its corresponding counterpart ${\hat{z}}_{x}$:

(10) $$L_{x}=E\left[{∥z_{x}-{\hat{z}}_{x}∥}_{2}^{2}\right]$$

where *x* ∈ {onset-id, onset-motion, apex-id, apex-motion} corresponds to the feature pairs detailed earlier: $z_{\mathrm{onset}-\mathrm{id}}$ and ${\hat{z}}_{\mathrm{onset}-\mathrm{id}}$ denote the identity features of $I_{\mathrm{onset}}$ and $I_{\mathrm{onset}\to \mathrm{apex}}$ (both extracted by $E_{\mathrm{id}}$); $z_{\mathrm{onset}-\mathrm{motion}}$ and ${\hat{z}}_{\mathrm{onset}-\mathrm{motion}}$ denote the motion features of $I_{\mathrm{apex}}$ and $I_{\mathrm{onset}\to \mathrm{apex}}$ (both extracted by $E_{\mathrm{motion}}$); and the symmetric pairs for $I_{\mathrm{apex}\to \mathrm{onset}}$ follow the same logic.

#### 3.2.5. Identity-Aware Contrastive Constraint

Given identity features $E_{\mathrm{id}}\left(I\right)$ extracted by the identity encoder, we first normalize them to unit vectors:

(11) $$\hat{s}=\frac{E_{\mathrm{id}}\left(I\right)}{∥E_{\mathrm{id}}{\left(I\right)∥}_{2}}$$

For a batch of features $\left\{{\hat{s}}_{i}\right\}$ with identity labels $\left\{y_{i}\right\}$, we define the angular distance between pairs as follows:

(12) $$d_{i,j}=\arccos\left({\hat{s}}_{i}\cdot {\hat{s}}_{j}^{⊤}\right)$$

**Figure 4:**
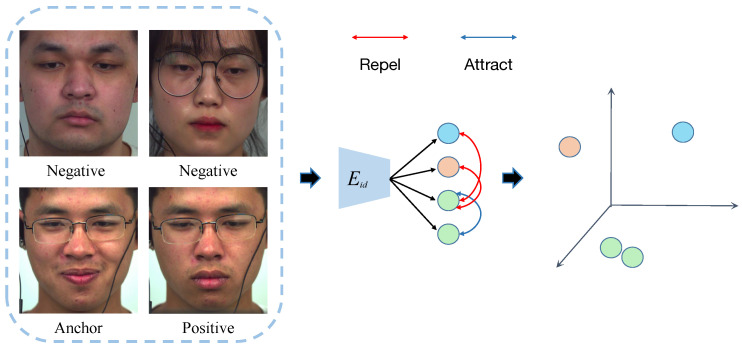
Identity-aware contrastive loss.

The identity loss is formulated as follows:

(13) $$L_{\mathrm{id}}=\frac{1}{\left|P\right|}\sum_{(i,j)\in P}d_{i,j}^{2}+\frac{1}{\left|N\right|}\sum_{(i,j)\in N}\max{\left(0,\alpha -d_{i,j}\right)}^{2}$$

where $P=\{\left(i,j\right)|y_{i}=y_{j},i<j\}$ and $N=\{\left(i,j\right)|y_{i}\neq y_{j},i<j\}$ denote sets of positive (same identity) and negative (different identity) pairs, |·| is the set size, and α=0.7 is a margin. As shown in Figure 4, this loss strengthens the consistency of identity features across original and synthetic images, ensuring they encode stable individual traits while being further separated from motion features.

The primary goal of the identity-aware contrastive loss is to reduce identity-induced variability, allowing the model to better focus on subtle emotion-related motion cues that are key for micro-expression recognition. Stabilizing identity features helps disentangle motion features from identity and improves emotion classification accuracy.

#### 3.2.6. Emotion Classification Constraint

To ensure that the disentangled representations are effectively aligned with emotion semantics, we employ the standard cross-entropy loss to supervise emotion prediction, formulated as follows:

(14) $$L_{\mathrm{emo}}=-\frac{1}{N}\sum_{i=1}^{N}\sum_{c=1}^{C}y_{i,c}\log\left(p_{i,c}\right)$$

where *N* denotes the batch size, *i* indexes each sample in the batch, *C* represents the number of emotion categories, $y_{i,c}$ is the one-hot encoded ground-truth label, and $p_{i,c}$ is the predicted probability of class *c* obtained through a softmax layer.

### 3.3. Temporal-Complemented Motion Enhancement Module

Micro-expressions involve extremely subtle and transient facial muscle movements, which cannot be sufficiently captured by static spatial representations alone. To model both spatial motion structure and dynamic motion continuity, we design a TCME module. It consists of a motion branch that learns spatial deformation between the onset and apex frames, and a complementary optical-flow-based branch that encodes fine-grained temporal variations. By integrating these two heterogeneous yet complementary motion cues, the TCME module generates a more discriminative spatiotemporal representation for MER.

#### 3.3.1. Motion Branch

**Figure 5:**
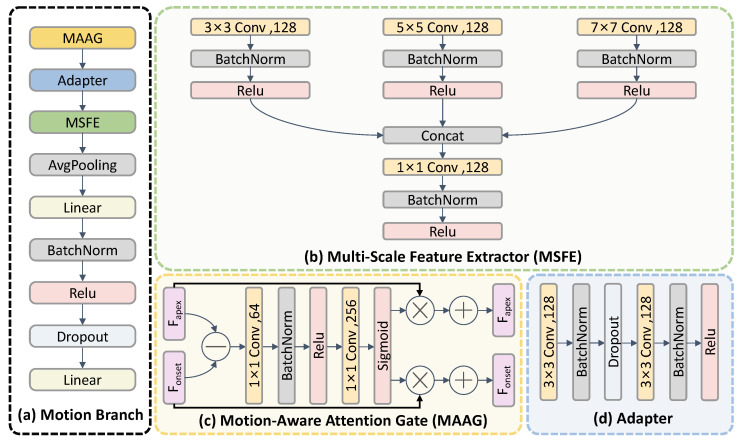
(**a**) is the overall pipeline of the motion branch, (**b**) is the Multi-Scale Feature Extractor (MSFE) module, (**c**) is the Motion-Aware Attention Gate (MAAG) module, and (**d**) is the Adapter module.

The motion branch focuses on modeling spatial motion structure by capturing local geometric deformations between the onset and apex frames. These deformations reflect subtle facial muscle activations, which are key motion cues for micro-expression representation. To enhance discriminativeness, we design a Motion-Aware Attention Gate (MAAG), which selectively amplifies regions exhibiting noticeable motion changes, as shown in Figure 5c. $F_{\mathrm{onset}}=E_{\mathrm{motion}}\left(I_{\mathrm{onset}}\right)$ and $F_{\mathrm{apex}}=E_{\mathrm{motion}}\left(I_{\mathrm{apex}}\right)$ represent motion features from onset and apex frames. The MAAG first computes motion differences $d_{\mathrm{motion}}$ between features; the lightweight attention module then uses two 1 × 1 convolution, BatchNorm, ReLU, and Sigmoid activation to construct attention weights $A_{\mathrm{motion}}$; finally, feature enhancement is performed by weighting the features with attention weights $A_{\mathrm{motion}}$.

(15) $$d_{\mathrm{motion}}=F_{\mathrm{apex}}-F_{\mathrm{onset}}$$

(16) $$A_{\mathrm{motion}}=\mathrm{Attention}(|d_{\mathrm{motion}}\left|\right)$$

(17) $$\begin{array}{cc} {\tilde{F}}_{\mathrm{onset}} & =F_{\mathrm{onset}}\cdot \left(1+\alpha \cdot A_{\mathrm{motion}}\right) \end{array}$$

(18) $$\begin{array}{cc} {\tilde{F}}_{\mathrm{apex}} & =F_{\mathrm{apex}}\cdot \left(1+\alpha \cdot A_{\mathrm{motion}}\right) \end{array}$$

where $A_{\mathrm{motion}}$ represents the attention weights generated by the attention module, ${\tilde{F}}_{\mathrm{onset}}$, ${\tilde{F}}_{\mathrm{apex}}$ denote the motion-enhanced features after attention weighting, and α is a scaling factor that controls the strength of motion enhancement (set to 0.2 in our experiments).

We insert a Adapter module after MAAG, as shown in Figure 5d. The Adapter simply concatenates the enhanced onset and apex features along the channel axis and employs two 3×3 convolutions to remap the doubled channels back to the original width, producing a consolidated spatial representation for subsequent multi-scale analysis.

To enrich spatial motion structure, we further introduce a Multi-Scale Feature Extractor (MSFE) that captures motion patterns at different receptive fields using parallel convolution kernels of sizes 3×3, 5×5, and 7×7. The fused output is aggregated by a 1×1 convolution.

Finally, adaptive average pooling, flattening, and a fully connected projection are applied to produce a 256-dimensional motion representation, which provides spatial motion encoding for micro-expression recognition.

#### 3.3.2. Optical Flow Branch

The optical flow branch complements the motion branch by introducing explicit temporal modeling. While the motion branch learns spatial deformation patterns between the onset and apex frames, the optical flow branch focuses on capturing continuous motion trajectories through optical flow, which describes pixel-level displacement over time. This explicit temporal cue enables the model to characterize fine-grained dynamic changes that spatial features alone cannot fully capture.

To model temporal motion cues, we compute TV-L1 optical flow [40] between onset and apex frames, obtaining horizontal flow *U*, vertical flow *V*, and optical strain OS that reflects local muscle deformation. These three components are stacked into a normalized tensor of size $R^{28\times 28\times 3}$.

We build a Triple Stream InceptionNet (TSI) to model *U*, *V*, and OS independently. Specifically, each optical flow component is converted into a 3-channel RGB image and fed into the corresponding stream (We also tried a single-stream architecture to model the temporal features of the 3-channel optical flow, but it achieved inferior performance; detailed results are provided later). Each stream employs two consecutive micro-inception blocks [41] to capture multi-scale temporal patterns through parallel convolution branches (1 × 1, 3 × 3, 5 × 5). Between the two micro-inception blocks, a spatial attention module and a channel attention module are inserted to adaptively emphasize motion-relevant information and suppress noise. Finally, the three temporal descriptors are concatenated and projected into a 256-dimensional temporal motion representation, which is later fused with the spatial motion features from the motion branch for MER classification.

## 4. Experiments

### 4.1. Databases

We conducted extensive experiments on the two recently publicly available large-scale datasets, CAS(ME)^3^ [42] and DFME [43]. The task-specific details of both datasets are shown in the Table 1.

CAS(ME)^3^ Part A comprises 860 samples from 100 participants. The video recordings, captured at 30 fps with a resolution of 1280 × 720, are categorized into four emotional categories: negative, positive, surprise, and others.

The largest ME dataset, DFME, contains 7526 samples from 656 people. This dataset was collected using high-speed cameras to capture ME segments at 500, 300, and 200 fps. Emotional labels for happiness, disgust, contempt, surprise, fear, anger, sadness, and others are annotated on each sample. Our experimental configuration uses the publicly accessible training set (1856 samples), test set A (474 samples), and test set B (299 samples) of this dataset.

### 4.2. Evaluation Protocols and Metrics

Following the original dataset study and earlier research, we use the Leave-One-Subject-Out (LOSO) cross-validation approach to assess performance on the CAS(ME)^3^ dataset. Concretely, in each iteration, all samples from an individual are assigned to the testing set and the rest to the training set. For the DFME dataset, we comply with the evaluation methodologies provided in [44], carrying out trials utilizing pre-divided training and testing subsets.

Due to the intrinsic class imbalance in most ME datasets, especially 7-class classification tasks, our model evaluation uses Unweighted F1-score (UF1) and Unweighted Average Recall (UAR) to assess performance fairly. For class *c*, let ${\mathrm{TP}}_{c}$, ${\mathrm{FP}}_{c}$, and ${\mathrm{FN}}_{c}$ denote true positives, false positives, and false negatives. The relevant calculations are as follows:

(19) $$UF1=\frac{1}{C}\sum_{i=1}^{C}\frac{2\cdot {\mathrm{Precision}}_{c}\cdot {\mathrm{Recall}}_{c}}{{\mathrm{Precision}}_{c}+{\mathrm{Recall}}_{c}},$$

where the precision and recall for class *c* are defined as follows:

(20) $${\mathrm{Precision}}_{c}=\frac{{\mathrm{TP}}_{c}}{{\mathrm{TP}}_{c}+{\mathrm{FP}}_{c}},$$

(21) $${\mathrm{Recall}}_{c}=\frac{{\mathrm{TP}}_{c}}{{\mathrm{TP}}_{c}+{\mathrm{FN}}_{c}}.$$

The UF1 metric computes the average F1-score across all classes without any weighting based on class occurrence frequency.

(22) $$\mathrm{UAR}=\frac{1}{C}\sum_{i=1}^{C}{\mathrm{Recall}}_{c}.$$

UAR works by averaging the recall values of each class, treating every class with equal importance, irrespective of how many samples each class contains.

### 4.3. Configuration

All experiments are conducted using PyTorch version 1.13.0, and the model training and inference are executed on a single NVIDIA GeForce RTX 4090 GPU.

### 4.4. Training Details

We adopt a three-stage training strategy that progressively builds feature disentanglement capabilities.

Stage 1 focuses on learning disentangled representations by freezing the TCME module gradients. The encoders and generator work together to establish robust feature separation and reconstruction abilities. We optimize the following objective:

(23) $$L_{\mathrm{stage}1}=\alpha_{1}L_{\mathrm{ort}}+\alpha_{2}L_{\mathrm{consist}}+\alpha_{3}L_{\mathrm{rec}}+\alpha_{4}L_{\mathrm{cyc}}+\alpha_{5}L_{\mathrm{id}}$$

We set $\alpha_{1}=0.3$, $\alpha_{2}=0.5$, $\alpha_{3}=20.0$, $\alpha_{4}=10.0$, $\alpha_{5}=5.0$, with perceptual loss weight β=0.05 and identity margin γ=0.7. The high reconstruction weights ($\alpha_{3}$, $\alpha_{4}$) ensure quality image generation, while smaller orthogonality weights ($\alpha_{1}$) provide gentle disentanglement guidance.

Stage 2 shifts focus to emotion classification by freezing the encoder-generator components and training only the TCME module. This preserves the learned feature separation while developing classification capabilities:

(24) $$L_{\mathrm{stage}2}=L_{\mathrm{emo}}$$

Using emotion loss solely prevents interference with established disentanglement and allows the classifier to learn effective motion-to-emotion mapping.

Stage 3 performs fine-tuning with all components active. This balances feature disentanglement with classification refinement:

(25) $$L_{\mathrm{stage}3}=\alpha_{1}L_{\mathrm{ort}}+\alpha_{2}L_{\mathrm{consist}}+\alpha_{3}L_{\mathrm{rec}}+\alpha_{4}L_{\mathrm{cyc}}+\alpha_{5}L_{\mathrm{id}}+\alpha_{6}L_{\mathrm{emo}}$$

We use $\alpha_{1}=0.2$, $\alpha_{2}=0.2$, $\alpha_{3}=10.0$, $\alpha_{4}=5.0$, $\alpha_{5}=1.0$, $\alpha_{6}=5.0$. The reduced reconstruction weights compared to stage 1 accommodate emotion loss while maintaining disentanglement quality.

### 4.5. Comparison to State-of-the-Art Methods

Previous approaches struggled with the complex samples of CAS(ME)^3^, making it one of the most challenging datasets. Table 2 presents performance metrics for various approaches. The proposed model enhances UF1 and UAR performance by 32.98% and 33.78% compared to AlexNet, a traditional deep learning approach. This significant improvement demonstrates that the MER network needs a more task-relevant module design. RCN, a standard deep learning method for MER, addresses domain shift in composite-database MER through model and data shrinking strategies. By comparison, our proposed method enhances UF1 by 19.40% and UAR by 21.19%. The proposed method outperforms HTNet, a current state-of-the-art approach using a hierarchical Transformer for local feature learning via self-attention, by 1.01% in UF1 and 5.97% in UAR. It also outperforms Micro-BERT by 2.64% in UF1, minimizing misclassifications, though it lags slightly by 1.13% in UAR. This trade-off highlights the proposed method’s superior MER categorization quality.

DFME is the largest publicly available ME dataset and poses significant challenges. Table 3 presents the seven-category verification performance on both test sets A and B. On test A, our approach outperforms the CCAC2024 DFME Challenge champion [44] by 1.37% in UF1 and 0.51% in UAR, and also surpasses the state-of-the-art HTNet. It is encouraging to see that our method works best on test B as well, where it beats the best rival (HTNet) by 0.37% in UF1 and 1.26% in UAR. These results show that the DRL and TCME module work well together to capture spatiotemporal ME patterns, which makes cross-subject generalization easier on the challenging DFME dataset.

### 4.6. Ablation Experiments

#### 4.6.1. DRL Module Ablation Study Analysis

Table 4 displays our model’s performance across various settings of the DRL module. In setting I (without ID Encoder and Generator), the model only has UF1 0.5409 and UAR 0.5563. To recognize MEs, the model uses only the motion encoder to extract motion structural features, combined with temporal information from the optical flow branch. The reason for the lower performance may be that the motion features are fully coupled with the identification features, and the feature disentanglement network degenerates into a conventional network structure. In Setting II, we implemented an identity feature encoder to better separate the motion structure from the identity features. Experimental results show that extracting identification and motion structure characteristics independently improves the network’s understanding of face muscle structures and motion patterns, reducing feature coupling issues. However, we still cannot guarantee that the identity and motion structure features understood by the network align with our human perception of these features. Therefore, we use a generator in Setting III to perform the facial reconstruction task. The result shows that our feature disentanglement network effectively distinguishes between identity and motion structure features and achieves the best performance.

#### 4.6.2. Optical Flow Branch Structure Analysis

Table 5 lists the comparative experiment with triple stream and single stream optical flow branch network structures. The results show that the triple stream Inceptionnet structure outperforms the single stream structure. From the perspective of network structure, the triple stream design allows for modality-specific, interference-free feature learning via parameter decoupling; In contrast, the single stream structure combines different motion cues into one input, which forces the shared convolutional kernels to learn a set of filters that mix horizontal, vertical, and strain patterns. This causes cross-modal interference and lowers the quality of the representation.

#### 4.6.3. TCME Module Ablation Study Analysis

Table 6 lists our model’s performance across various settings of TCME module. In Setting I, the model does not incorporate the motion branch and optical flow branch, and only uses features from motion encoders to perform MER. Results indicate that while the model can discern the motion structure of onset and apex frames, it fails to capture temporal relationships or uncover muscle movement patterns that genuinely reflect emotional states. Setting II incorporates a motion branch. The motion branch did a good job of finding essential parts of the face and modeling spatial motion patterns. This is proven by the fact that UF1 and UAR went up by 4.05% and 5.58%, respectively, compared to Setting II. Setting III adds an optical flow branch, which makes the system work at its best. The TSI network we built adds different points of view to the motion branch, which improves the entire model.

#### 4.6.4. Multi-Loss Function Ablation Experiment

Table 7 displays our model’s performance across various loss function settings. In Setting II, the soft orthogonality loss $L_{\mathrm{ort}}$ enhances UF1 and UAR by 1.36% and 1.54%, respectively, compared to Setting I. The soft orthogonality loss improves motion structure feature purity, reducing subject-relevant features and improving generalization efficiency. In Setting III, the cycle reconstruction loss $L_{\mathrm{cyc}}$ improves the model’s ability in reconstructing original images compared to Setting II, which greatly promotes the model’s understanding of subtle motion cues to avoid over-decoupling; therefore, UF1 and UAR increased by 0.58% and 0.50% respectively. Setting IV incorporates consistency loss $L_{\mathrm{consist}}$. Despite its small performance influence, it helps the model discover stable micro-expression movement patterns and improves training stability. By adding identity loss $L_{\mathrm{id}}$, Setting V achieves optimal performance metrics, indicating that improving identity features also improves motion structure understanding.

### 4.7. Interpretability Analysis

**Figure 6:**
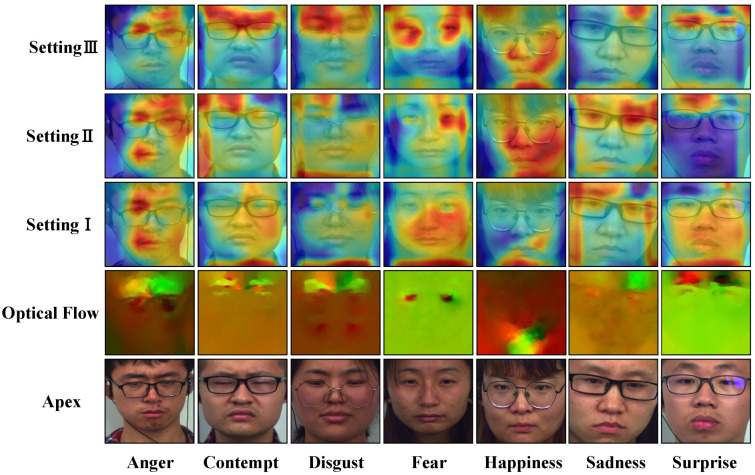
Model attention visualization based on Grad-CAM, Setting consistent with the Table 6’s Setting.

We provide Grad-CAM [54] attention visualizations for the model, as illustrated in Figure 6, following the module ablation experiment parameters in Table 6. Each column represents a fundamental emotion. The visualizations for each row, from bottom to top, are the apex frame, optical flow (as an auxiliary reference for motion occurrence location), setting I using only the motion encoder, setting II using both the motion and identity encoders, and setting III using the entire network architecture. From the visualization results, we can see that the model pays more attention to the local areas where the movement occurs (such as the eyes and mouth) rather than the location that reflects the identity information. This also proves our point: based on SDO joint optimization, the DRL module effectively separates motion information irrelevant to identity, and the TCME module further focuses on purified spatial motion cues and fuses them with the temporal dynamics brought by the optical flow branch to provide a comprehensive spatiotemporal motion representation of MEs.

**Figure 7:**
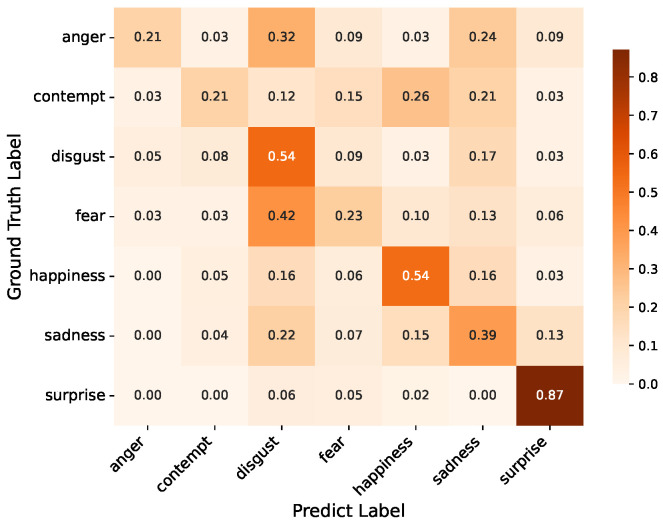
Confusion matrix on DFME test A.

**Figure 8:**
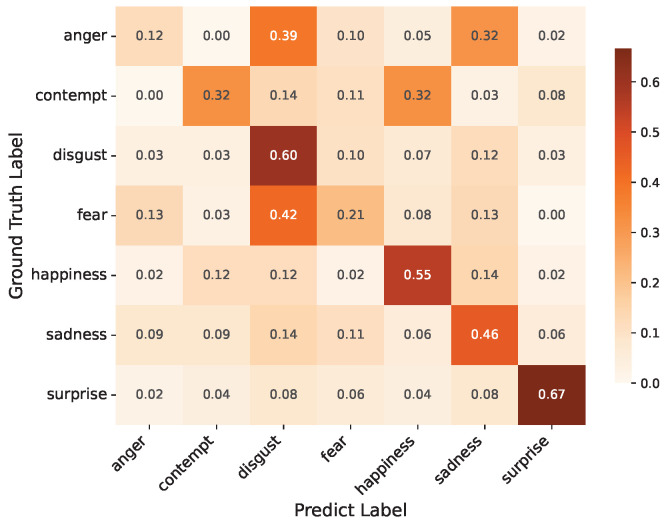
Confusion matrix on DFME test B.

**Figure 9:**
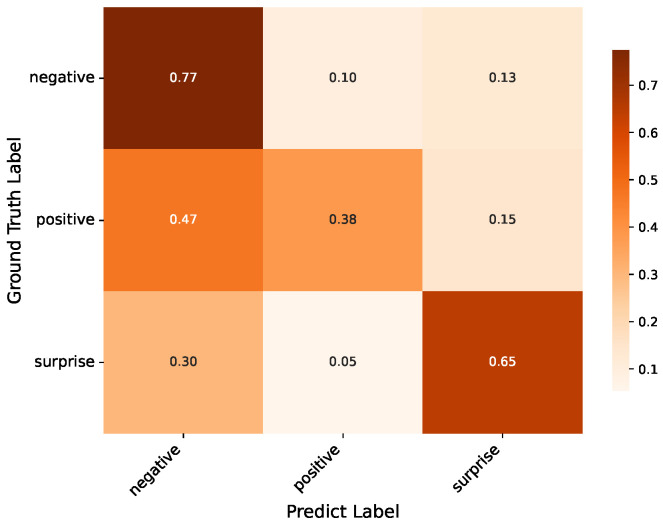
Confusion matrix on CAS(ME)^3^ part A.

Additionally, we provide confusion matrices on the DFME test sets A/B and CAS(ME)^3^ for further analysis, as shown in Figure 7, Figure 8 and Figure 9. The analysis reveals distinct performance patterns across different emotional categories and datasets.

On the DFME Test A dataset (7-class classification), our model exhibits remarkable proficiency in recognizing surprise, achieving an accuracy rate of 87%. This outstanding performance stems from the distinct spatiotemporal patterns of surprise—characterized by abrupt eye widening, eyebrow raising, and mouth opening—which generate prominent motion cues that are easily distinguishable from other emotions. These motion features, even in subtle ME forms, maintain strong uniqueness, enabling the model’s TCME module to effectively capture and amplify such discriminative signals. The model achieves a respectable 54% accuracy for happiness and disgust. Happiness MEs usually involve subtle zygomaticus major muscle activation (i.e., mouth corner lifting). Disgust, characterized by slight nose wrinkling and upper lip raising, also presents localized, recognizable motion cues. These motions maintain consistent local patterns that are more easily separable from identity features, thereby supporting the model’s moderate performance. In contrast, anger and contempt achieve only 21% accuracy, the lowest among all categories. This is mainly because the model’s capacity to learn stable motion patterns is limited by the small number of training samples (39 anger and 34 contempt samples in Test A). Additionally, their MEs are extremely subtle—anger often involves faint brow furrowing or jaw tightening, while contempt manifests as a subtle unilateral lip curl—lacking distinctive spatial signatures that are easily distinguishable from other negative emotions (e.g., anger is frequently misclassified as disgust), leading to low recognition accuracy.

The DFME Test B dataset presents a slightly different performance profile compared to Test A. Despite a noticeable 20-percentage-point decrease from Test A, surprise is still the most accurately identified emotion with 67% accuracy. This is mostly because its samples were drastically reduced (from 101 in Test A to 48 in Test B, a decrease of more than 50%). Even though surprise has unique motion characteristics by nature, the model’s generalization is limited by the small sample size, which causes the accuracy to decline. Notably, disgust maintains stable performance at approximately 60%, verifying the robustness of the model’s feature extraction for this emotion. Happiness maintains a stable accuracy of 55%, despite the reduced sample size (from 63 to 42 samples in Test B). This stability underscores the model’s robustness in capturing happiness’s consistent motion cues—even with fewer samples, the distinctive and localized nature of these motions allows the model to retain reliable recognition performance. In Test B, anger has the lowest accuracy of 12%. The confusion matrix reveals significant misclassification as disgust (39%) and sadness (32%), despite the fact that its sample count (41) is comparable to Test A’s (39). This is due to its minor, overlapping motion signals with other unpleasant emotions.

The model shows different behavioral patterns for the CAS(ME)^3^ Part A dataset, where we conduct three-class classification experiments (negative, positive, and surprise). Negative emotions (a composite category encompassing anger, disgust, fear, and sadness) achieve the highest recognition rate of 77%. This strong performance benefits from the cumulative sample size of negative emotions (457 samples), providing the model with sufficient training data to learn shared negative emotion motion characteristics (e.g., generalized facial muscle tension). Additionally, the DRL module effectively isolates these shared motion features from identity information, enhancing the model’s ability to generalize across different negative sub-emotions. Surprise follows with a 65% recognition rate, consistent with its strong performance in DFME datasets—its unique and intense motion pattern (eye and mouth opening) remains highly discriminative even in the 3-class setting. However, positive emotions achieve only 38% accuracy, the lowest among the three categories. The confusion matrix indicates that 47% of positive samples are misclassified as negative, primarily due to two factors: first, the severe class imbalance (only 55 positive samples versus 457 negative samples) leads the model to favor the majority negative class during prediction; second, positive MEs (subtle mouth corner lifting) exhibit weaker motion intensity compared to negative emotions or surprise, making their motion features prone to being overwhelmed by identity-related information or misjudged as subtle negative expressions (e.g., a slight smile may be confused with a neutral or faintly sad expression). Notably, 15% of positive samples are misclassified as surprise, which may stem from partial overlap in facial muscle activation—both emotions can involve subtle mouth movements, leading to confusion when motion cues are not sufficiently distinct.

The experiments’ results on all three datasets highlight some essential factors that affect MER performance. First, surprise consistently achieves a relatively high recognition rate across datasets, as its facial movements (e.g., eye widening, brow raising) possess inherently unique and distinguishable spatiotemporal characteristics. Second, dataset imbalance significantly impacts model performance—emotions with scarce training samples (e.g., positive emotions in CAS(ME)^3^, anger and contempt in DFME) often suffer from insufficient feature learning and high misclassification rates. Third, individual differences in emotional expression (e.g., varying ME amplitudes and movement patterns across subjects) add inherent complexity to MER tasks. These findings underscore the imperative of addressing dataset balance, mitigating feature variability, and accommodating expression amplitude inconsistencies in developing robust MER systems.

### 4.8. Model Complexity Analysis

Table 8 compares the model complexity of LFD-TCMEN variants with representative optical-flow-based micro-expression recognition methods. Without the optical flow branch, LFD-TCMEN contains 5.49 M parameters, while the proposed triple-stream optical flow version contains 7.92 M parameters. Despite this increase, the overall complexity remains within the range of mainstream CNN-based architectures that explicitly incorporate optical flow for MER, and is notably lower than Transformer-based designs such as HTNet. These results show that incorporating the optical flow branch increases the model complexity from 5.49 M to 7.92 M parameters, while maintaining a parameter scale comparable to existing optical-flow-based CNN methods.

## 5. Discussion

The experimental findings support our central hypothesis that learnable feature disentanglement—which draws inspiration from the Bruce–Young model of facial cognition—offers a more effective paradigm for micro-expression recognition than conventional fixed preprocessing techniques.

The most significant finding is that end-to-end disentanglement substantially improves cross-subject generalization. Our DRL module learns to adaptively isolate pure motion patterns during training, in contrast to RPCA-based approaches that handle identity-motion separation as a disconnected preprocessing step. This benefit is supported by the ablation results in Table 6, which shows that identity encoding and reconstruction gradually enhance performance, with the complete architecture obtaining a 4.59% higher UF1 than motion-only encoding. This supports the biological plausibility of our design: our dual-encoder architecture learns distinct representations that more accurately capture emotion-relevant motion cues, just as the human brain processes facial identity and expression through functionally independent neural pathways.

Equally important is the role of the SDO scheme in preventing over-disentanglement. A significant issue in feature separation is the possible loss of nuanced motion data crucial for MER. The multi-loss ablation in Table 7 shows that each constraint has a real effect: the reconstruction loss keeps motion fidelity, the soft orthogonality loss makes sure that features are independent, and the identity-aware contrastive loss makes identity encoding stronger without affecting motion representations. This synergistic optimization addresses the fundamental tension between disentanglement strength and motion preservation.

The confusion matrices in Figure 7, Figure 8 and Figure 9 further reveal how our approach handles different emotion categories. The consistently high recognition rates for surprise (65–87% across datasets) demonstrate that the TCME module effectively captures distinctive spatiotemporal patterns—the abrupt eye widening and mouth opening generate prominent motion cues that benefit from both spatial attention (MAAG) and optical flow temporal modeling. However, emotions with subtle, ambiguous motion signatures (anger, contempt, and fear) remain challenging, as their micro-movements exhibit low inter-class discriminability—the confusion matrices show frequent misclassification among these negative emotions due to overlapping facial muscle activation patterns. These patterns suggest that while learnable disentanglement effectively addresses identity-motion entanglement, the inherent discriminability of different emotion categories poses additional challenges that warrant future investigation through class-balanced strategies or multi-modal fusion.

## 6. Conclusions

In MER, the entanglement between emotion-related motion features and identity-specific appearance severely limits cross-subject generalization. Building on the Bruce–Young model of facial cognition, which provides a theoretical basis for the independent processing of identity and expression, we introduce LFD-TCMEN, a novel learnable feature disentanglement network that establishes the first end-to-end disentanglement paradigm for MER. The proposed DRL module separates purified motion patterns from appearance features, while the TCME module enriches spatial motion features with optical-flow-based temporal dynamics to strengthen subtle motion perception and enable precise spatiotemporal modeling. Moreover, the entire network is optimized under the SDO scheme, which harmonizes multiple complementary losses to jointly guide feature disentanglement and temporal representation learning. This multi-constraint scheme ensures that the disentanglement process effectively isolates identity features while preserving critical motion information, preventing excessive separation that could result in the loss of subtle motion cues essential for accurate MER. Extensive experiments on CAS(ME)^3^ and DFME demonstrate that LFD-TCMEN achieves new state-of-the-art performance in cross-subject MER, validating the effectiveness of our learnable disentanglement, synergistic optimization, and motion enhancement design. Despite these achievements, our method has several limitations. First, the recognition accuracy for emotions with subtle motion patterns (e.g., anger, contempt, and fear) remains limited due to their low inter-class discriminability and insufficient training samples. Second, our framework relies on accurate onset-apex frame selection, which may introduce errors in real-world scenarios where apex frames are not readily available. Finally, while our method demonstrates strong cross-subject generalization within datasets, cross-database evaluation remains unexplored, and the varying recording conditions across different ME databases may pose additional challenges. In future work, we plan to address these limitations by incorporating class-balanced learning strategies or multi-modal fusion to improve the recognition of subtle emotions, integrating automatic apex frame detection to reduce reliance on manual keyframe annotation, and conducting cross-database experiments with domain adaptation techniques to enhance generalization across diverse recording conditions.

## Figures and Tables

**Table 1:** Statistics on task settings and data distribution for DFME and CAS(ME)^3^.

Dataset	Task	Total	Distribution of Labels
CAS(ME)^3^	3-class (part A)	699	Negative (457) Positive (55) Surprise (187)
DFME	7-class (train)	1856	Anger (161) Contempt (100) Disgust (548) Fear (265) Happiness (206) Sadness (278) Surprise (298)
	7-class (test A)	474	Anger (39) Contempt (34) Disgust (129) Fear (62) Happiness (63) Sadness (46) Surprise (101)
	7-class (test B)	299	Anger (41) Contempt (37) Disgust (58) Fear (38) Happiness (42) Sadness (35) Surprise (48)

The Negative category combines Anger, Disgust, Fear, and Sadness. The Positive category consists of Happy.

**Table 2:** SOTA methods comparison on CAS(ME)^3^ dataset. The symbol ↑ indicates that higher values represent better performance. Bold font denotes the best performance, and underlined values indicate the second-best performance.

Methods	Year	UF1 ↑	UAR ↑
AlexNet [45]	2012	0.2570	0.2634
STSTNet [9]	2019	0.3795	0.3792
RCN-A [46]	2020	0.3928	0.3893
MERSiam [47]	2021	0.3184	0.3532
FGRL [11]	2021	0.3333	0.2636
FR [48]	2022	0.3493	0.3413
MMNet [49]	2022	0.3706	0.3646
BDCNN [28]	2022	0.5050	0.5164
Micro-BERT [50]	2023	0.5604	**0.6125**
HTNet [15]	2024	0.5767	0.5415
FAMNet [51]	2025	0.4342	0.5100
**Ours**	-	**0.5868**	0.6012

**Table 3:** SOTA methods comparison on DFME dataset. The symbol ↑ indicates that higher values represent better performance. Bold font denotes the best performance, and underlined values indicate the second-best performance.

Methods	Year	Test Set	UF1 ↑	UAR ↑
FR [48]	2022		0.3410	0.3686
Wang et al. [44]	2024		0.4067	0.4074
He et al. [44]	2024		0.4123	0.4210
HTNet [15]	2024	Test A	0.3736	0.3821
MambaVision-B [52]	2024		0.4002	0.4064
MELLM [53]	2024		0.3578	0.3732
**Ours**	-		**0.4260**	**0.4261**
FR [48]	2022		0.2875	0.3228
Wang et al. [44]	2024		0.3534	0.3661
He et al. [44]	2024		0.4016	0.4008
HTNet [15]	2024	Test B	0.4076	0.4062
MambaVision-B [52]	2024		0.3929	0.3858
MELLM [53]	2024		0.3162	0.3424
**Ours**	-		**0.4113**	**0.4188**

**Table 4:** Ablation experiment of DRL module on CAS(ME)^3^ dataset. The symbol ↑ indicates that higher values represent better performance. Bold font denotes the best performance.

Setting	Motion Encoder	ID Encoder	Generator	UF1 ↑	UAR ↑
I	✓			0.5409	0.5563
II	✓	✓		0.5699	0.5817
III	✓	✓	✓	**0.5868**	**0.6012**

**Table 5:** Comparative experiment with triple-stream and single-stream optical flow branch network structures. The symbol ↑ indicates that higher values represent better performance. Bold font denotes the better performance.

Setting	UF1 ↑	UAR ↑
Single Stream	0.5654	0.5817
Triple Stream	**0.5868**	**0.6012**

**Table 6:** Ablation experiment of TCME module on CAS(ME)^3^ dataset. The symbol ↑ indicates that higher values represent better performance. Bold font denotes the best performance.

Setting	Motion Branch	Optical Flow Branch	UF1 ↑	UAR ↑
I			0.5034	0.5076
II	✓		0.5439	0.5634
III	✓	✓	**0.5868**	**0.6012**

**Table 7:** Loss ablation study on CAS(ME)^3^ dataset. The symbol ↑ indicates that higher values represent better performance. Bold font denotes the best performance.

Setting	$L_{\mathrm{rec}}$	$L_{\mathrm{ort}}$	$L_{\mathrm{cyc}}$	$L_{\mathrm{consist}}$	$L_{\mathrm{id}}$	UF1 ↑	UAR ↑
I	✓					0.5574	0.5725
II	✓	✓				0.5710	0.5879
III	✓	✓	✓			0.5768	0.5929
IV	✓	✓	✓	✓		0.5780	0.5936
V	✓	✓	✓	✓	✓	**0.5868**	**0.6012**

**Table 8:** Model complexity comparison in terms of the number of parameters. The symbol # denotes the number of model parameters, measured in millions (M).

Method	#Params (M)
STSTNet	0.0017
BDCNN	6.56
Feature Refinement (FR)	10.90
HTNet	140.63
LFD-TCMEN (w/o OF)	5.49
LFD-TCMEN (Single-OF)	6.30
LFD-TCMEN (Triple-OF, Ours)	7.92

## Data Availability

The micro-expression data required for this work can be obtained through the following website: https://mea-lab-421.github.io/ (accessed on 25 January 2026) for the DFME dataset, and http://casme.psych.ac.cn/ (accessed on 25 January 2026) for the CAS(ME)^3^ dataset.
